# Machine learning application for the prediction of SARS-CoV-2 infection using blood tests and chest radiograph

**DOI:** 10.1038/s41598-021-93719-2

**Published:** 2021-07-09

**Authors:** Richard Du, Efstratios D. Tsougenis, Joshua W. K. Ho, Joyce K. Y. Chan, Keith W. H. Chiu, Benjamin X. H. Fang, Ming Yen Ng, Siu-Ting Leung, Christine S. Y. Lo, Ho-Yuen F. Wong, Hiu-Yin S. Lam, Long-Fung J. Chiu, Tiffany Y So, Ka Tak Wong, Yiu Chung I. Wong, Kevin Yu, Yiu-Cheong Yeung, Thomas Chik, Joanna W. K. Pang, Abraham Ka-chung Wai, Michael D. Kuo, Tina P. W. Lam, Pek-Lan Khong, Ngai-Tseung Cheung, Varut Vardhanabhuti

**Affiliations:** 1 Department of Diagnostic Radiology, Li Ka Shing Faculty of Medicine, The University of Hong Kong, Hong Kong, SAR China; 2 Artificial Intelligence Lab, Head Office Information Technology and Health Informatics Division, Hospital Authority, Hong Kong, SAR China; 3 The School of Biomedical Sciences, Li Ka Shing Faculty of Medicine, The University of Hong Kong, Hong Kong, SAR China; 4 Clinical Systems, Information Technology and Health Informatics Division, Hospital Authority, Hong Kong, SAR China; 5 Department of Radiology, Queen Mary Hospital, Hong Kong, SAR China; 6 Department of Medical Imaging, The University of Hong Kong-Shenzhen Hospital, Shenzhen, China; 7 Department of Radiology, Pamela Youde Nethersole Eastern Hospital, Hong Kong, SAR China; 8 Department of Radiology, Hong Kong Sanatorium & Hospital, Hong Kong, SAR China; 9 Department of Radiology and Imaging, Queen Elizabeth Hospital, Hong Kong, SAR China; 10 Department of Imaging and Interventional Radiology, Faculty of Medicine, The Chinese University of Hong Kong, Hong Kong, China; 11 Department of Imaging and Interventional Radiology, Prince of Wales Hospital, Hong Kong, SAR China; 12 Department of Radiology, Tuen Muen Hospital, Hong Kong, SAR China; 13 Department of Medicine, Princess Margaret Hospital, Hong Kong, SAR China; 14 Health Informatics, Information Technology and Health Informatics Division, Hospital Authority, Hong Kong, SAR China; 15 Emergency Medicine Unit, Li Ka Shing, Faculty of Medicine, The University of Hong Kong, Hong Kong, China; 16 Information Technology and Health Informatics Division, Hospital Authority, Hong Kong, SAR China

**Keywords:** Computational biology and bioinformatics, Biomarkers, Health care, Mathematics and computing

## Abstract

Triaging and prioritising patients for RT-PCR test had been essential in the management of COVID-19 in resource-scarce countries. In this study, we applied machine learning (ML) to the task of detection of SARS-CoV-2 infection using basic laboratory markers. We performed the statistical analysis and trained an ML model on a retrospective cohort of 5148 patients from 24 hospitals in Hong Kong to classify COVID-19 and other aetiology of pneumonia. We validated the model on three temporal validation sets from different waves of infection in Hong Kong. For predicting SARS-CoV-2 infection, the ML model achieved high AUCs and specificity but low sensitivity in all three validation sets (AUC: 89.9–95.8%; Sensitivity: 55.5–77.8%; Specificity: 91.5–98.3%). When used in adjunction with radiologist interpretations of chest radiographs, the sensitivity was over 90% while keeping moderate specificity. Our study showed that machine learning model based on readily available laboratory markers could achieve high accuracy in predicting SARS-CoV-2 infection.

## Introduction

Since being declared a global pandemic on 11th March 2020, the infection of severe acute respiratory syndrome coronavirus 2 (SARS–CoV-2), known officially as COVID-19, has rapidly spread globally. Multiple waves of infections have been observed in several countries around the world, and despite efforts in mass vaccination, this is likely to take some time to get the viruses fully under control at a global level. We also have to combat the possibility of perpetually recurring waves of infection as the world battles against the emergence of variants. Therefore, it still remains of paramount importance to be able to provide a timely diagnosis to the different affected regions with scalability.

Reverse transcriptase-polymerase chain reaction (RT-PCR) tests, although regarded as the gold standard, has reported false-negative rates being variably quoted between 10–61%^1,2^. There is also a disparity in testing capability globally. In western countries such as Europe and North America, the cumulative number of tests per population is 10 times that of Asia and 34 times that of Africa as of the end of August 2020^3^. In resource-scarce settings, substitute tests may be needed to prioritise RT-PCR for vulnerable or high risk group. Early reports have shown that there are important characteristics in laboratory blood results such as leucopenia and lymphopenia^4–7^. Several prior studies have assessed the utility of non-specific inflammatory biomarkers such as C-reactive protein (CRP), white cell count (WBC) and absolute neutrophil count (ANC) to discriminate probable bacterial infections from non-bacterial infections^8,9^. Still, as of yet, none have examined these in context with COVID-19 infection. Hong Kong also offers a unique perspective in this regard in being affected at a relatively early stage from a global perspective with initial outbreaks coinciding with local seasonal influenza infections. Several studies have examined descriptive characteristics of COVID-19 laboratory markers^4,5,10^, but machine learning applications offer another potential way to incorporate more subtle relationships between different laboratory markers^11^. A few studies have recently been published regarding the use of machine learning for diagnosis. For example, Zoabi et al. (2021) applied machine learning technique for prediction of COVID-19 using eight clinical and demographics binary features^12^. There is also a potential adjunct role of imaging in aiding the diagnosis of COVID-19. Chest radiographic abnormalities have been reported at the initial presentation of COVID-19^5,13,14^ is more scalable/readily available compared to CT and, has been advocated as a radiology decision tool for suspected COVID-19 by the British Society of Thoracic Imaging^15^.

The objective of this study is to apply machine learning for the task of COVID-19 detection using basic laboratory markers and explore the adjunctive role of chest radiographs. Here, we initially performed a statistical comparison of blood tests in patients with different aetiologies of pneumonia, including COVID-19 involving 5,148 patients in 24 hospitals in Hong Kong during the first and second waves of infection. This is to establish a baseline laboratory comparison between COVID-19 from other pneumonia and other diagnoses. We then trained and validated machine learning models using basic blood tests with comparison to reference RT-PCR testing to predict COVID-19 infection status, and explore different use case scenarios with adjunction of chest radiographs. The models were then validated with temporal validation sets across other waves of infection in Hong Kong.

## Results

### Patient cohorts and analysis

#### Primary cohort

**Figure 1:**
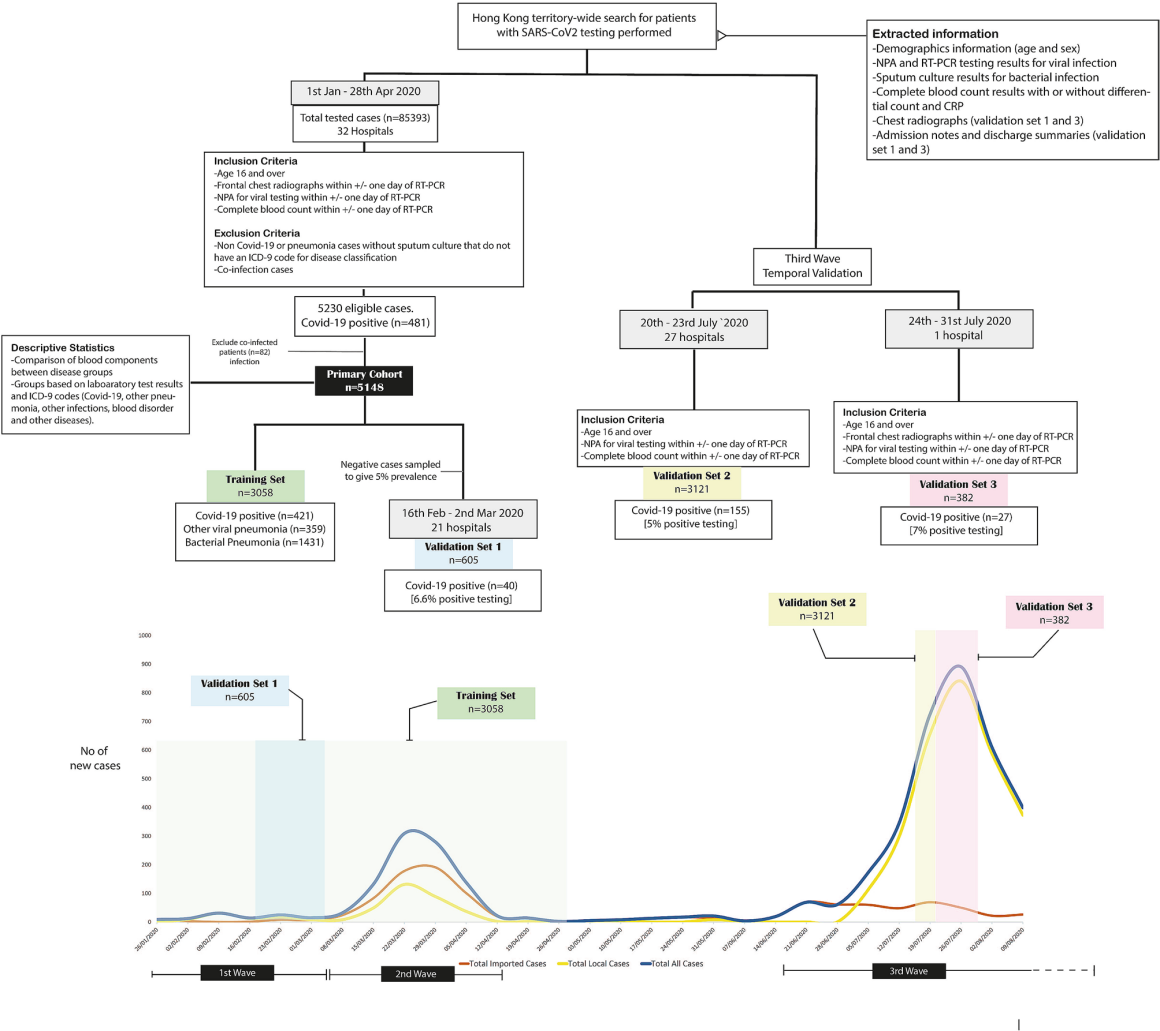
Schematic showing study design with patient selection at each point of the study and temporal representation of training and validation sets in Hong Kong.

Summary of the study design and local outbreak timeline is presented in Fig. 1. From the start of the local outbreak to 28th April 2020, a total of 85,393 patients from 32 hospitals in Hong Kong had taken the RT-PCR test for SARS-CoV-2 virus. After applying the inclusion and exclusion criteria, a total of 5230 patients were eligible and included in the primary cohort. Of the 5230 patients, 18 (0.3%) patients were co-infected with COVID-19 and bacterial pneumonia, 15 (0.3%) patients were co-infected with COVID-19 and another viral infection, 48 (0.9%) patients were co-infected with bacterial and non-COVID-19 viral pneumonia, and one patient was coinfected with all three. Due to the low amount of cases, the coinfected cases were removed from further analysis (n = 82). The primary cohort then finally included a total of 5148 patients. Of these, 447 patients were COVID-19 (8.7%), 405 patients (7.9%) with other viral pneumonia, and 1515 patients (29.4%) with bacterial pneumonia. A total of 1,862 (36.2%) were classified as clinical pneumonia with no laboratory confirmation or incomplete tests. For the non-pneumonia patient, there were 919 patients (17.96%), of whom 256 (5.0%) were classified with other (non-pneumonia) infections by ICD-9 classification. Baseline characteristics of the primary cohort with laboratory tests and differences between disease groups are described in Table 1.

**Table 1:** Baseline demographics and laboratory characteristics of the primary cohort.

	COVID-19	Other viral PNA	Bacterial PNA	Clinical PNA	Other infections	Other diseases
**Demographics**						
Total, n	447	405	1515	1862	256	663
Female, n (%)	202 (45)	192 (47)	570 (38)	865 (46)	138 (54)	276 (42)
**Age, years**						
Mean ± SD	42 ± 17	53 ± 22	74 ± 17	73 ± 19	45 ± 21	61 ± 21
Median (IQR)	39 (28–57)	52 (35–70)	77 (65–87)	79 (62–88)	39 (29–59)	64 (46–79)
16–35	174 (39)	101 (25)	46 (3)	117 (6)	104 (41)	105 (16)
35–50	113 (25)	87 (21)	85 (6)	147 (8)	62 (24)	82 (12)
50–65	115 (26)	83 (20)	237 (16)	281 (15)	37 (14)	152 (23)
> 65	45 (10)	134 (33)	1147 (76)	1317 (71)	53 (21)	324 (49)
**Haemoglobin g/dL**						
Missing, n (%)	0 (0)	0 (0)	0 (0)	0 (0)	0 (0)	0 (0)
Mean ± SD	13.9 ± 1.4	12.9 ± 2.2	11.1 ± 2.4	11.4 ± 2.5	13.2 ± 2.0	12.0 ± 2.6
Median (IQR)	13.9 (13.0–15.0)	13.2 (11.7–14.6)	11.1 (9.3–12.8)	11.7 (9.7–13.2)	13.4 (12.1–14.5)	12.4 (10.3–14.0)
**Haematocrit**						
Missing, n (%)	0 (0)	0 (0)	0 (0)	1 (0)	0 (0)	0 (0)
Mean ± SD	0.4 ± 0.0	0.4 ± 0.1	0.3 ± 0.1	0.3 ± 0.1	0.4 ± 0.1	0.4 ± 0.1
Median (IQR)	0.4 (0.4–0.4)	0.4 (0.4–0.4)	0.3 (0.3–0.4)	0.4 (0.3–0.4)	0.4 (0.4–0.4)	0.4 (0.3–0.4)
**WBC, 10**^**9**^**/L**						
Missing, n (%)	0 (0)	0 (0)	0 (0)	0 (0)	0 (0)	0 (0)
Mean ± SD	5.5 ± 1.9	8.8 ± 4.8	12.6 ± 12.9	11.3 ± 6.7	9.4 ± 5.5	11.5 ± 21.5
Median (IQR)	5.2 (4.3–6.4)	7.7 (6.0–10.2)	11.1 (7.5–15.4)	9.8 (7.1–13.7)	7.7 (6.3–10.5)	9.0 (6.8–12.4)
**Lymphocyte, 10**^**9**^**/L**						
Missing, n (%)	4 (1)	48 (12)	97 (6)	108 (6)	17 (7)	43 (6)
Mean ± SD	1.3 ± 0.6	1.4 ± 0.8	1.1 ± 2.1	1.2 ± 1.5	1.7 ± 1.1	1.4 ± 2.5
Median (IQR)	1.3 (0.9–1.7)	1.3 (0.8–1.8)	0.9 (0.5–1.4)	1.0 (0.6–1.5)	1.6 (1.0–2.1)	1.2 (0.7–1.7)
**Monocyte 10**^**9**^**/L**						
Missing, n (%)	4 (1)	48 (12)	100 (7)	108 (6)	17 (7)	44 (7)
Mean ± SD	0.5 ± 0.2	0.6 ± 0.4	0.7 ± 1.0	0.8 ± 1.8	0.6 ± 0.4	0.7 ± 0.9
Median (IQR)	0.5 (0.3–0.6)	0.6 (0.4–0.8)	0.6 (0.4–0.9)	0.6 (0.4–0.9)	0.5 (0.3–0.7)	0.6 (0.4–0.9)
**Neutrophil 10**^**9**^**/L**						
Missing, n (%)	4 (1)	48 (12)	97 (6)	108 (6)	17 (7)	43 (6)
Mean ± SD	3.6 ± 1.7	6.7 ± 4.8	10.4 ± 7.4	9.1 ± 5.5	7.1 ± 5.6	8.4 ± 5.7
Median (IQR)	3.3 (2.4–4.4)	5.4 (3.9–7.8)	8.9 (5.8–13.5)	7.8 (5.1–11.6)	5.2 (3.9–8.3)	6.7 (4.5–10.6)
**Platelet 10**^**9**^**/L**						
Missing, n (%)	0 (0)	0 (0)	4 (0)	2 (0)	0 (0)	0 (0)
Mean ± SD	221.2 ± 74.1	233.3 ± 87.4	246.0 ± 117.7	241.2 ± 108.7	234.1 ± 81.2	255.4 ± 108.4
Median (IQR)	205.0 (171.0–259.0)	223.0 (173.0–280.0)	231.0 (169.5–307.0)	224.5 (168.0–294.0)	233.0 (179.0–281.2)	236.0 (188.0–301.0)
**CRP mg/dL**						
Missing, n (%)	48 (11)	181 (45)	610 (40)	638 (34)	122 (48)	292 (44)
Mean ± SD	1.9 ± 4.0	4.9 ± 7.7	9.9 ± 9.6	7.4 ± 8.1	5.7 ± 9.4	5.9 ± 8.0
Median (IQR)	0.4 (0.2–1.4)	1.9 (0.4–6.0)	7.1 (2.1–15.2)	4.9 (1.1–11.0)	0.7 (0.1–7.0)	2.1 (0.3–8.8)
**LDH U/L**						
Missing, n (%)	31 (7)	250 (62)	936 (62)	1020 (55)	146 (57)	347 (52)
Mean ± SD	208.9 ± 74.8	238.1 ± 124.7	371.9 ± 625.5	287.0 ± 344.9	198.5 ± 73.4	296.8 ± 500.6
Median (IQR)	186.5 (158.0–237.0)	210.0 (165.0–253.2)	252.6 (193.7–353.0)	226.5 (184.0–290.0)	177.0 (155.5–212.0)	210.5 (178.0–293.2)

*SD* standard deviation, *IQR* intequartile range, *PNA* pneumonia, *WBC* white blood cell, *CRP* C-reactive protein, *LDH* lactate dehydrogenase.

**Figure 2:**
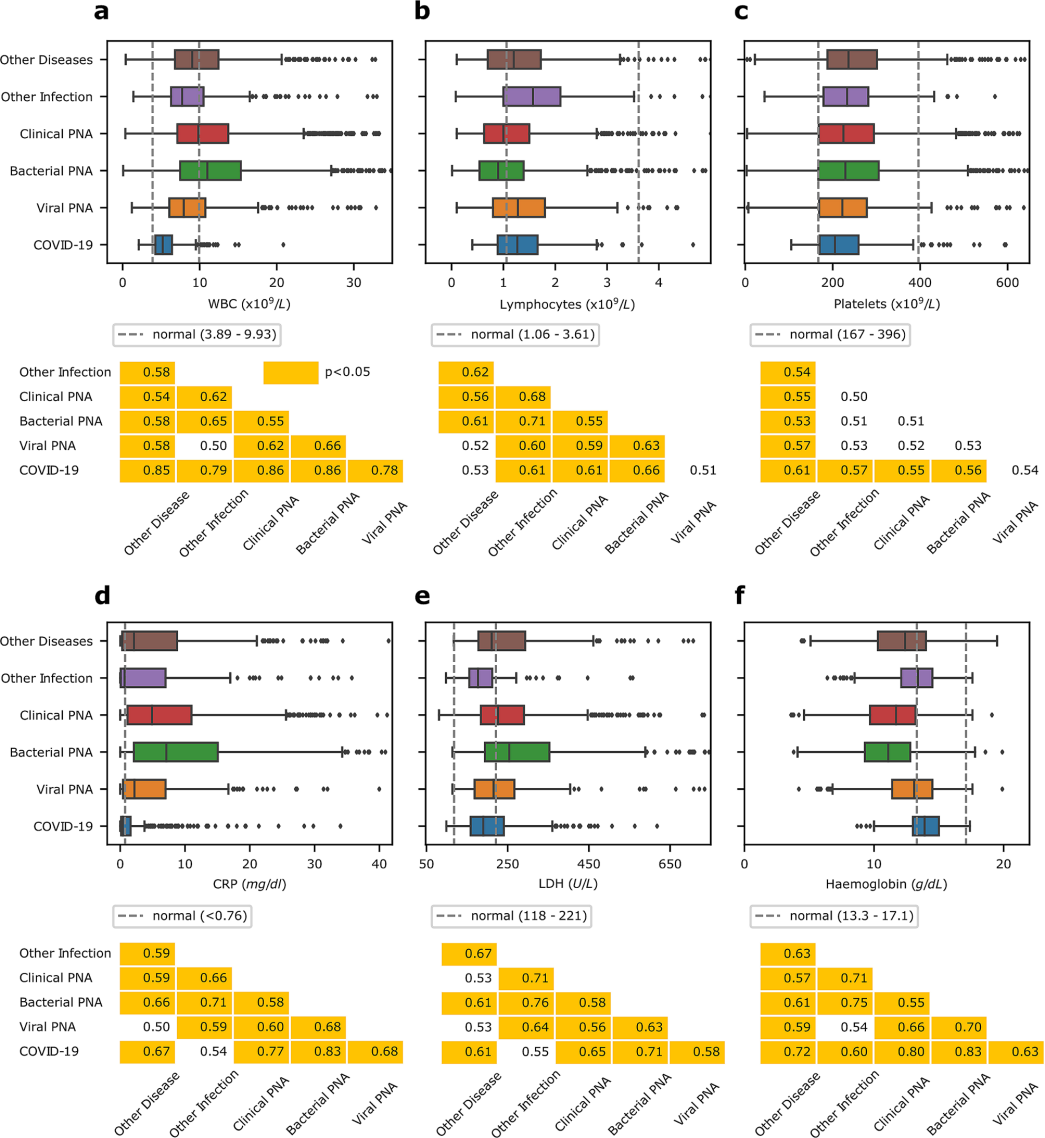
Box Plots and pairwise Mann–Whitney U test summary for common blood laboratory markers. For each blood laboratory marker, the lower and upper bounds of the diagnostic reference range adopted in the local hospitals are given by the grey dotted lines. Statistical significance is indicated by the orange highlights, and the effect size estimated by f is given in the table. If statistical significance is achieved this is highlighted in orange. **(a)** Boxplot for comparing white blood cell (WBC) counts across different disease groups (Kruskal–Wallis H: p < 0.001). **(b)** Boxplot for comparing lymphocyte counts across different disease groups (Kruskal–Wallis H: p < 0.001). **(c)** Boxplot for comparing platelet counts across different disease groups (Kruskal–Wallis H: p < 0.001). **(d)** Boxplot for comparing C-reactive protein (CRP) level across different disease groups (Kruskal–Wallis H: p < 0.001). **(e)** Boxplot for comparing lactate dehydrogenase (LDH) level across different disease groups (Kruskal–Wallis H: p < 0.001). **(f)** Boxplot for comparing haemoglobin distribution across different disease groups (Kruskal–Wallis H: p < 0.001). *PNA* pneumonia.

There were significant differences between patient age across disease groups (Kruskal–Wallis H: p < 0.001). Patients with COVID-19 were the youngest and were significantly younger than other viral (Mann–Whitney: p < 0.001) and bacterial pneumonia (Mann–Whitney: p < 0.001). Box plots describing the distribution of the laboratory blood markers are presented in Fig. 2. WBC was significantly lower in patients with COVID-19 than any other disease groups with large estimated effect sizes (f = 0.78 to 0.86). CRP and LDH were also found to be statistically lower in COVID-19 patients compared to other groups except for other non-pneumonia infections. In contrast, WBC, CRP and LDH were found to be highest in bacterial pneumonia.

Correlation between each laboratory markers and age was analysed. Neutrophils count was found to be highly positively correlated with WBC (r_s_ = 0.96; p < 0.001). In addition, monocytes and WBC were found to be moderately correlated (r_s_ = 0.53; p < 0.001). Haemoglobin were also found to be highly correlated with haematocrit (r_s_ = 0.98; p < 0.001), and moderately correlated with age (r_s_ = 0.45; p < 0.001). No other features were found to be moderately or strongly correlated with age (r_s_ = -0.30 to 0.28).

#### Validation cohorts

To evaluate the performance of the discriminative model, three validation cohorts across different periods of the epidemic in Hong Kong were obtained. Baseline demographics and clinical characteristics comparing COVID-19 and non-COVID-19 patients in the validation sets are presented in Table 2. A total of 605 patients were obtained for validation set 1, of whom 40 patients were positive for COVID-19. A subset of patients in validation set 1 that fulfilled the criteria for the primary cohort was obtained to test the performance of the model for detecting other subtypes of pneumonia. Distribution of laboratory markers between subtypes of pneumonia of the validation set 1 are given in Supplementary Table 1. Validation set 2 and 3 were consecutive temporal validation sets based on patients that falls outside period of the primary cohort. As the time of the validation set 2 and 3 was outside of influenza season, many of the patients were only tested for a subset of common viruses (Viral group 1 in Supplementary Fig. 1). Of those patients who had viral testing performed, only four patients have confirmed positive in the validation set 3, and no patients in the validation set 2. Due to the low number of confirmed cases, model performance for pneumonia subtype was not assessed in validation sets 2 and 3.

**Table 2:** Baseline demographics and laboratory and clinical characteristics of validation sets.

	Validation set 1		Validation set 2^a^		Validation set 3	
	COVID-19	Non COVID-19	COVID-19	Non COVID-19	COVID-19	Non COVID-19
**Demographics**						
Total, n	40	565	155	2966	27	355
Female, n (%)	25 (62)	261 (46)	84 (54)	1420 (48)	15 (56)	181 (51)
**Age, years**						
Mean ± SD	57 ± 19	67 ± 20	54 ± 17	67 ± 20	55 ± 19	72 ± 19
Median (IQR)	58 (46–70)	70 (54–85)	56 (40–66)	71 (55–84)	58 (43–66)	78 (61–87)
16–35	7 (18)	48 (8)	30 (19)	267 (9)	5 (19)	20 (6)
35–50	6 (15)	74 (13)	30 (19)	328 (11)	5 (19)	36 (10)
50–65	11 (28)	105 (19)	51 (33)	590 (20)	9 (33)	48 (14)
> 65	16 (40)	338 (60)	44 (28)	1781 (60)	8 (30)	251 (71)
**Haemoglobin g/dL**						
Missing, n (%)	0 (0)	0 (0)	5 (3)	15 (1)	1 (4)	5 (1)
Mean ± SD	12.7 ± 1.9	11.7 ± 2.5	13.5 ± 1.5	12.0 ± 2.5	13.2 ± 2.0	11.5 ± 2.6
Median (IQR)	12.8 (11.3–14.2)	11.8 (9.9–13.3)	13.4 (12.7–14.5)	12.2 (10.4–13.7)	13.5 (13.0–14.3)	11.6 (9.8–13.4)
**Haematocrit**						
Missing, n (%)	0 (0)	0 (0)	6 (4)	119 (4)	1 (4)	5 (1)
Mean ± SD	0.4 ± 0.1	0.4 ± 0.1	0.4 ± 0.0	0.4 ± 0.1	0.4 ± 0.1	0.3 ± 0.1
Median (IQR)	0.4 (0.3–0.4)	0.4 (0.3–0.4)	0.4 (0.4–0.4)	0.4 (0.3–0.4)	0.4 (0.4–0.4)	0.3 (0.3–0.4)
**WBC, 10**^**9**^**/L**						
Missing, n (%)	0 (0)	0 (0)	6 (4)	119 (4)	1 (4)	5 (1)
Mean ± SD	6.7 ± 2.4	10.7 ± 6.0	5.3 ± 1.7	10.3 ± 6.0	5.0 ± 1.5	10.6 ± 5.7
Median (IQR)	6.2 (5.1–8.0)	9.2 (6.7–13.0)	5.0 (4.1–6.2)	9.0 (6.6–12.5)	4.7 (3.9–6.0)	9.4 (7.0–13.0)
**Lymphocyte, 10**^**9**^**/L**						
Missing, n (%)	0 (0)	0 (0)	8 (5)	618 (21)	1 (4)	6 (2)
Mean ± SD	1.4 ± 0.6	1.2 ± 0.8	1.2 ± 0.5	1.4 ± 0.9	1.1 ± 0.5	1.3 ± 0.8
Median (IQR)	1.3 (0.9–1.7)	1.0 (0.7–1.6)	1.2 (0.9–1.6)	1.2 (0.8–1.8)	1.0 (0.8–1.3)	1.2 (0.8–1.7)
**Monocyte 10**^**9**^**/L**						
Missing, n (%)	0 (0)	0 (0)	8 (5)	618 (21)	1 (4)	6 (2)
Mean ± SD	0.5 ± 0.2	0.7 ± 0.8	0.5 ± 0.2	0.7 ± 0.5	0.6 ± 0.3	0.7 ± 0.4
Median (IQR)	0.5 (0.4–0.7)	0.6 (0.4–0.9)	0.5 (0.4–0.7)	0.6 (0.4–0.8)	0.5 (0.4–0.7)	0.6 (0.5–0.9)
**Neutrophil 10**^**9**^**/L**						
Missing, n (%)	0 (0)	0 (0)	8 (5)	617 (21)	1 (4)	5 (1)
Mean ± SD	4.6 ± 2.4	8.5 ± 5.5	3.5 ± 1.6	8.0 ± 5.6	3.2 ± 1.2	8.2 ± 4.9
Median (IQR)	4.2 (2.9–5.2)	7.0 (4.7–10.7)	3.1 (2.3–4.2)	6.6 (4.4–10.3)	3.1 (2.2–3.7)	7.0 (4.7–10.2)
**Platelet 10**^**9**^**/L**						
Missing, n (%)	0 (0)	0 (0)	6 (4)	122 (4)	1 (4)	6 (2)
Mean ± SD	255.0 ± 101.9	241.5 ± 105.6	198.2 ± 59.5	247.2 ± 100.2	202.7 ± 49.4	245.5 ± 107.2
Median (IQR)	238.0 (171.8–318.5)	232.0 (172.0–297.0)	193.0 (156.0–239.0)	233.5 (183.0–294.0)	187.0 (173.2–229.8)	238.0 (184.0–291.0)
**CRP mg/dL**						
Missing, n (%)	1 (2)	223 (39)	35 (23)	1642 (55)	8 (30)	311 (88)
Mean ± SD	4.1 ± 7.4	8.2 ± 9.6	2.3 ± 3.5	6.1 ± 7.3	1.9 ± 2.7	3.2 ± 3.8
Median (IQR)	0.7 (0.3–2.4)	4.4 (0.9–11.9)	0.7 (0.3–2.9)	3.3 (0.6–8.8)	0.5 (0.4–2.1)	1.8 (0.5–4.3)
**LDH U/L**						
Missing, n (%)	4 (10)	365 (65)	63 (41)	2501 (84)	4 (15)	341 (96)
Mean ± SD	245.3 ± 79.4	274.5 ± 145.4	227.4 ± 121.5	287.4 ± 339.4	251.3 ± 80.6	527.1 ± 769.1
Median (IQR)	228.0 (189.5–268.0)	226.0 (185.8–322.8)	190.2 (172.8–235.0)	225.0 (182.0–290.0)	231.0 (193.0–286.0)	280.5 (195.2–442.0)
**Contact history**						
Travel, n (%)	12 (30)	133 (24)	N/A	N/A	0 (0)	2 (1)
Close contact, n (%)	20 (50)	14 (2)	N/A	N/A	19 (70)	6 (2)
**Symptoms**						
Fever, n (%)	19 (48)	254 (45)	N/A	N/A	15 (56)	168 (47)
Cough, n (%)	23 (58)	295 (52)	N/A	N/A	16 (59)	92 (26)
URTI, n (%)	14 (35)	94 (17)	N/A	N/A	14 (52)	33 (9)
Shortness of breath, n (%)	10 (25)	236 (42)	N/A	N/A	1 (4)	122 (34)
Headache, n (%)	2 (5)	16 (3)	N/A	N/A	4 (15)	19 (5)
Myalgia, n (%)	3 (8)	9 (2)	N/A	N/A	0 (0)	85 (24)
Nausea & vomiting, n (%)	1 (2)	26 (5)	N/A	N/A	0 (0)	85 (24)
Diarrhoea, n (%)	1 (2)	20 (4)	N/A	N/A	4 (15)	36 (10)
Anosmia, n (%)	0 (0)	0 (0)	N/A	N/A	5 (19)	2 (1)
**Admission condition**						
Fever (> 37.5 °C), n (%)	9 (22)	111 (20)	N/A	N/A	20 (74)	166 (47)
Requiring supplemental oxygen, n (%)	4 (10)	140 (25)	N/A	N/A	2 (7)	85 (24)

*SD* standard deviation, *IQR* interquartile range, *WBC* white blood cell, *CRP* C-reactive protein, *LDH* lactate dehydrogenase.

^a^Clinical characteristics were not extracted for validation set 2.

### Development of a machine learning model to detect COVID-19 and other subtypes of pneumonia

Driven by the observation of primary cohort analysis and to further analyse the discriminability of basic laboratory markers, a machine learning classifier was trained to classify whether the patient has COVID-19, other viral pneumonia, bacterial pneumonia or non-pneumonia. A total of 3,058 patients from the primary cohort was used as the training set. Of these, 421 patients (13.8%) were COVID-19 confirmed, 359 patients (11.7%) were of other viral pneumonia, 1431 patients (46.8%) were of bacterial pneumonia, and 847 (27.7%) were of other diseases. Baseline characteristics of the primary cohort and laboratory tests of the training set are summarised in the Supplementary Table 2.

Given the significant differences in age between groups, to avoid bias, age and haemoglobin were not used for the model. In addition, monocytes, neutrophils and haematocrit were also removed for redundancy. The features selected for the final model were sex, WBC, lymphocytes, platelets, CRP and LDH. Several algorithms and classifiers were considered (see Supplementary Table 3). Categorical gradient boosting (CatBoost) was selected as the classifier of the model due to the ease of handling missing numbers and categorical features, and also produce the highest cross-validation performance. The CatBoost model was trained with 80% of the training set with the other 20% used for cross-validation, model selection, and threshold selection.

### Model evaluation

The performance of the ML model was validated on three validation sets. In addition, a clinical model was devised to provide baseline performance for the evaluation, along with radiologist interpretation. The clinical model was based on the early observation that lymphopenia associated with COVID-19. Local diagnostic ranges for lymphocytes were used for the model. The clinical model and radiologist interpretation were evaluated on the validation set 1 and 3. The performance of individual radiologist is presented in Supplementary Table 4.

The validation of all models in classifying COVID-19 is summarised in Table 3. For discriminating COVID-19, the ML model achieved high AUCs and specificity in all three validation sets (AUC > 0.9 and specificity > 0.9). Radiologists’ read achieved low sensitivity, and moderate to high specificity in the validation set 1 and set 3. When used together, the combined ML model and radiologists achieved a significantly higher sensitivity of over 90% in each validation sets but a reduction in specificity. The basic clinical model was not able to accurately identify COVID-19 patients. Performance of the model on the classification of other pneumonia subtypes in the validation set 1 is presented in Table 4. The model achieved a moderately high AUC of 77.4% in classifying bacterial pneumonia but was unable to adequately discriminate between other viral and non-pneumonia patients.

**Table 3:** COVID-19 discriminability of the machine learning model and comparison to clinical, radiologist consensus and combined model.

	Positive/total	AUC^a^	Accuracy	Sensitivity	Specificity	PPV	NPV
	n	% (95%-CI)	% (95%-CI)	% (95%-CI)	% (95%-CI)	% (95%-CI)	% (95%-CI)
**Validation set 1**							
ML model	40/605	89.9 (85.9–93.9)	89.3 (86.5–91.6)	57.5 (40.9–73.0)	91.5 (88.9–93.7)	32.6 (22.8–42.3)	97.9 (96.6–99.1)
Clinical model	40/605	N/A	70.4 (66.6–74.0)	30.0 (16.6–46.5)	73.3 (69.4–76.9)	7.4 (3.4–11.4)	93.7 (91.4–95.9)
Radiologist consensus	40/605	N/A	73.2 (69.5–76.7)	55.0 (38.5–70.7)	74.5 (70.7–78.1)	13.3 (8.1–18.4)	95.9 (94.0–97.8)
Radiologist + ML model	40/605	N/A	68.4 (64.6–72.1)	92.5 (79.6–98.4)	66.7 (62.7–70.6)	16.4 (11.6–21.3)	99.2 (98.3–100.1)
**Validation set 2**							
ML model	155/3121	91.3 (89.2–93.3)	93.0 (92.0–93.9)	57.4 (49.2–65.3)	94.8 (94.0–95.6)	36.8 (30.7–42.9)	97.7 (97.2–98.3)
**Validation set 3**							
ML model	27/382	95.8 (91.6–99.9)	96.9 (94.6–98.4)	77.8 (57.7–91.4)	98.3 (96.4–99.4)	77.8 (62.1–93.5)	98.3 (97.0–99.7)
Clinical model	27/382	N/A	67.2 (62.2–71.9)	57.7 (36.9–76.6)	67.9 (62.7–72.8)	11.8 (6.2–17.4)	95.6 (93.0–98.1)
Radiologist read^b^	27/382	N/A	92.3 (89.1–94.8)	53.8 (33.4–73.4)	95.1 (92.3–97.1)	45.2 (27.6–62.7)	96.5 (94.6–98.5)
Radiologist + ML model	27/382	N/A	55.5 (50.3–60.6)	92.3 (74.9–99.1)	52.7 (47.3–58.1)	12.7 (8.0–17.4)	98.9 (97.4–100.4)

*AUC* area under the curve, *PPV* positive predictive value, *NPV* negative predictive value, *CI* confidence intervals, *ML* machine learning model.

^a^AUC for Clinical, Radiologist and combined Radiologist and ML model are not applicable.

^b^For validation set 2, only one radiologist interpreted the chest radiograph for validation set 3.

**Table 4:** Pneumonia subtype discriminability of the machine learning model.

Disease	Positive/total	AUC, % (CI)	Accuracy, % (CI)	Sensitivity, % (CI)	Specificity, % (CI)
	N	% (95%-CI)	% (95%-CI)	% (95%-CI)	% (95%-CI)
Bacterial PNA	45/175	77.4 (70.2–84.5)	55.4 (47.7–62.9)	100.0 (92.1–100.0)	40.0 (31.5–49.0)
Viral PNA	49/175	62.9 (54.0–71.9)	56.6 (48.9–64.0)	67.3 (52.5–80.1)	52.4 (43.3–61.3)
Non PNA	62/175	62.5 (54.1–70.9)	61.7 (54.1–68.9)	38.7 (26.6–51.9)	74.3 (65.3–82.1)

*AUC* area under the curve, *PNA* pneumonia.

**Figure 3:**
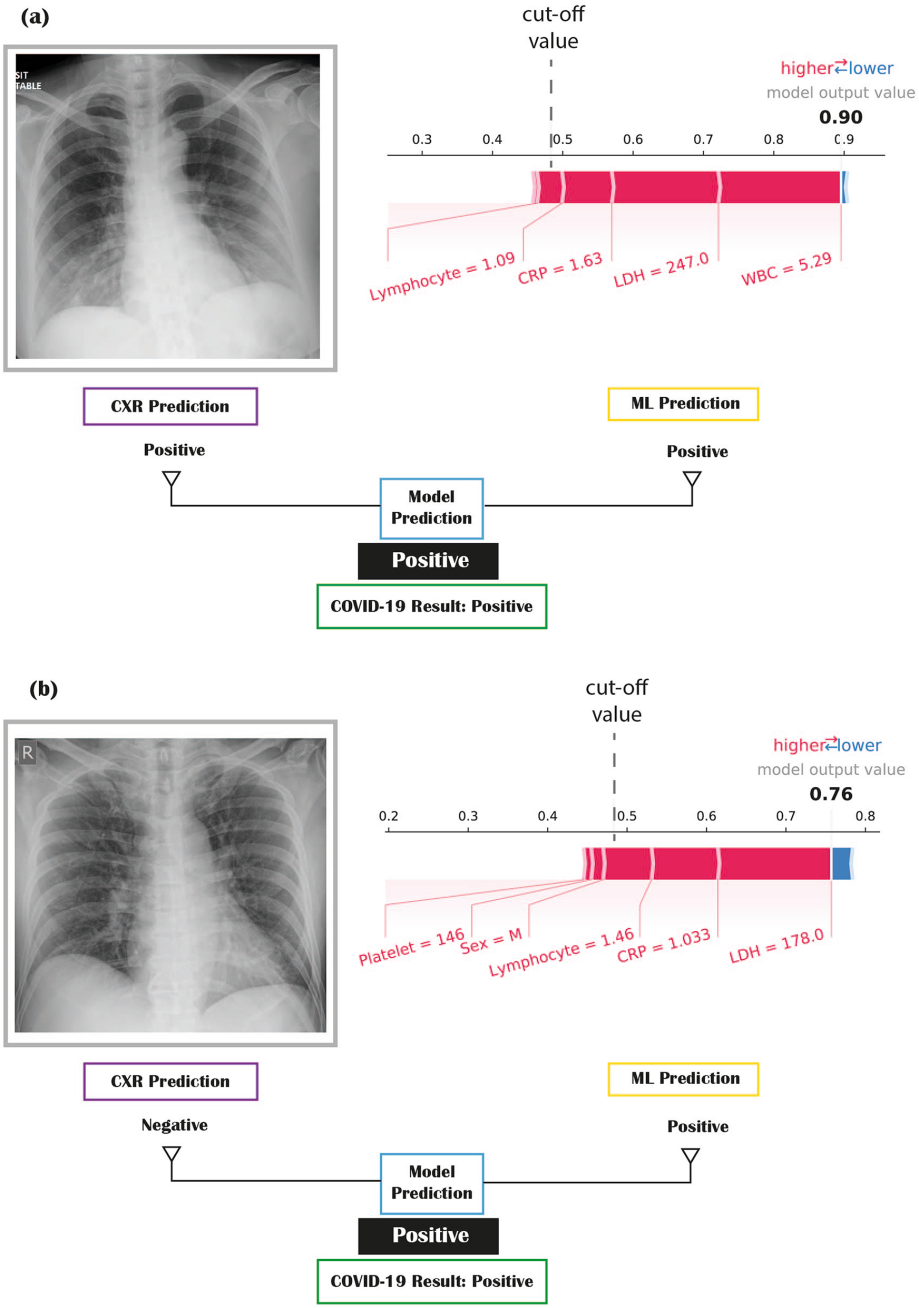
Case examples of human and machine learning model prediction. The cut-off threshold for SHAP model is 0.48 meaning that if the model output value is above this, then the prediction is positive. The relative contribution of each laboratory marker is shown in the individual SHAP value plot. **(a)** An elderly female with a positive prediction from chest X-ray (bilateral lower zones shadowing) and positive prediction from laboratory markers (WBC: 5.29, lymphocytes: 1.09, LDH: 247, and CRP: 1.63). The ground-truth COVID-19 RT-PCR result is positive. **(b)** An elderly male with a negative prediction from chest X-ray (normal radiographic appearance) and positive prediction from laboratory markers (LDH: 178, lymphocytes: 1.46, platelet: 146, and CRP: 1.033The ground-truth COVID-19 RT-PCR result is positive.

The SHAP analysis of the models shows that WBC was the most important predictor for COVID-19 with a decrease in WBC corresponding with a higher probability of COVID-19. For bacterial pneumonia, WBC and lymphocytes have the highest impact, with high WBC and low lymphocytes count corresponding to an increase in the likelihood of bacterial pneumonia. Summary plots for SHAP analysis and illustrative examples of how the final prediction using the combined model works in practice with the contribution of SHAP value are shown in Fig. 3 and supplementary Fig. 2.

## Discussion

There has been an emphasis on testing using RT-PCR in the early stages of management of the COVID-19 pandemic. Despite the growing availability of RT-PCR testing kits, confirmation is usually only available after triaging, or treatment decisions have been made. Leveraging existing infrastructures and differentiating from other common respiratory tract infections need to be considered for long term sustainability in combating the disease. There are two potential scenarios when using simple tests may be useful. First, a model may be helpful in countries that cannot afford large supplies of RT-PCR testing kits, particularly currently it is looking likely that the pandemic will assume a more protracted course with prolonged economic impact. Given the high sensitivity and negative predictive value of our combined model, it is potentially indicated for low-risk patient stratification, whereby a negative prediction from the ML model allows for patients’ discharge while awaiting final laboratory confirmation. The risk of subsequent community infection is thus minimised whilst not overburdening the healthcare system or isolation centres. Second, consider a scenario whereby the disease prevalence is low or becomes seasonal; the model may serve as a surveillance system for future outbreaks. The machine learning approach offers the potential of automation with tasks running in the background and only alerting clinicians in case of positive prediction. The tools being used here are based on clinical intuition. Using laboratory blood results for screening is already being done in clinical practice even at the early stages of the outbreak^16^. CXR radiographic appearances, although overlaps with other viral aetiologies^17^, when used in combination with blood test increases sensitivity. Machine learning has the potential to better handle non-linearly separable data thus achieving better performance. Despite that, analysis of our machine learning model had found linear association in some predictor such as WBC and CRP. WBC was significantly lower in COVID-19 patients than viral pneumonia patients, but the median value was still within the normal range. Human interpretation which relies solely on just the reference range may miss this subtlety.

Major strengths in our study include a large sample size of patients with reference laboratory testing in all cases, in a population where there was clinical suspicion of respiratory infection at the initial presentation. Our cohorts of positive COVID-19 cases were also consecutive during different phases of outbreaks in Hong Kong. The study also involved 27 hospitals in all territories of Hong Kong and was validated on three separate held-out test sets, with the latter two validation sets included consecutive patients during the third wave of infection. We also only used blood results and CXR at the initial presentation, which mirrors the potential use case. The COVID-19 cases in Hong Kong are unique as all the patients regardless of clinical severity were hospitalised. Our model is therefore likely applicable to patients with full disease spectrum.

Several recent studies have been published on COVID-19, but in the initial periods, these have mainly included clinical characteristics, laboratory findings, descriptive findings of radiological appearances and were mostly focusing on COVID-19 patients in isolation^5–7^. Our findings were broadly in line with previous studies with low white cell count and CRP having high discriminability. Of note, whilst the median lymphocyte count in our cohorts was low for COVID-19, it was similar to other viral pneumonia. It is known that other viral pneumonias were also associated with lymphopenia^8,18^. Moreover, the median value for non-pneumonia was even lower thus limiting its discriminating power. CRP in our cohort was raised but not as high compared to other pneumonia. Owing to different reference ranges, the actual values are not directly comparable with other studies. The findings may also reflect the range of clinical spectrum at presentations where our patients may present at an earlier stage compared to at the epicentre of the outbreak in other countries. Our CRP results are similar to one other territory-wide study that was performed in Hong Kong ^19^ and another smaller study from Taiwan^20^, which directly compared laboratory markers with other non-COVID-19 respiratory infections. This was also true in early-stage patients in a separate study^21^, as well as in one of the largest cohort to date which included severity of clinical status, where the CRP was higher in more severe groups reflecting more severe inflammatory states^5^. A few recent studies demonstrated the value in using data-driven machine learning approach in prognostication for COVID-19^22,23^, and have similarly identified lymphocytes and CRP to be important features, as well as LDH for predicting mortality. In terms of diagnostic capability with machine learning, some recent studies have also been performed, but with smaller datasets, lack of temporal validation and often without clinical comparison ^24–26^. More recently several machine learning based approaches have been published demonstrating more broader applicability in COVID-19 related applications including triage assessment^27^, severity classifcaiton^28,29^, risk prognostication including mortality^30^ as well as applying to multi-omics data^31^. For example, a similar approach was tried with similar findings also with an attempt for explanability similar to our study^32^. This study used decision trees and criteria graph whilst our study used SHAP analysis. Another recently published study also applied machine learning to clinical and laboratory improves the performance of the prediction of COVID-19^33^. There is increasing body of evidence in the literatures now supporting the potential usefulness in applying machine learning for these tasks.

Some limitations are worth noting. First, this is a retrospective study. Prospective validation of such models would be helpful to see how it performs in real practice. Second, there are potentially important features such as other laboratory and clinical features which were not used. Owing to the retrospective nature of this study, other blood tests were fewer in numbers in our cohorts. Clinical notes at the initial presentation were in hand-written formats and were not readily retrievable at scale across multiple hospitals for all patients. However, we were able to review these for validation sets 1 and 3. In particular, the duration of clinical symptoms may be helpful to include in future models as these may show better discriminability between seasonal influenza. Thirdly, the generalisability of the model needs to be tested in other settings. The sensitivity of any diagnostic test depends on patient characteristics. More specifically, predictive models are derived from the training datasets with its own distribution of disease severity and varying disease spectrum. In Hong Kong, all patients are admitted to hospitals or treatment centres regardless of their clinical status. Different countries have different approaches to testing and hospitalisation of patients with COVID-19, so the generalisability will depend on how well this matches with the idiosyncrasies of the individual healthcare practices.

In summary, a machine learning model was able to achieve high accuracy for the prediction of SARS-CoV-2 infection. Adjunctive use of chest radiograph could play a role in increasing sensitivity while achieving moderate specificity when combined with ML blood model, which may have potential implications in triaging patients, particularly when RT-PCR testing resources are scarce.

## Methods

### Ethics approval

This study protocol was approved by multi-institutional review boards in multiple hospitals across Hong Kong: HKU/Hong Kong West Cluster Research Ethics Committee (Ref. UW 20-291), Hong Kong East Cluster Research Ethics Committee (HKECREC-2020-012), Kowloon Central/Kowloon East Cluster Research Ethics Committee (KC/KE-20-0052/ER-3), Kowloon West Cluster Research Ethics Committee (Ref. KW/EX-20-065), CUHK/New Territories East Cluster Clinical Research Ethics Committee (Ref. 2020.216), and New Territories West Cluster Research Ethics Committee (NTWC/REC/20048). Informed patient consent was waived owing to the retrospective nature of the study. The study design followed the TRIPOD criteria^34^. For information, please refer to Supplementary Document. All methods were carried out in accordance with local authority guidelines and regulations. All experimental protocols were approved by a named institutional and/or licensing committee.

### Study design and cohort selections

The patients used in this study are based on a territory-wide search of patients with clinical suspicion of COVID-19 infection presenting to the accident and emergency department from the start of the COVID-19 outbreak. Patients that were retrieved had undergone RT-PCR testing for SARS-CoV-2 fulfilling the testing criteria by Centre for Health Protection, Department of Health, Government of Hong Kong SAR (see Supplementary document).

Due to a large number of patients who were screened because of cross-border travel or close contact with positive patients, to select symptomatic patients from the cohort, the following inclusion criteria were applied: (i) had frontal chest radiographs on the date of the RT-PCR test, (ii) had laboratory testing done, specifically haematological blood count with or without differential counts, C-reactive protein (CRP) and lactate dehydrogenase (LDH) on the date of the RT-PCR test. In addition to test results, the patient demographics and ICD diagnosis code at the date of the first examination of each patient were also retrieved. Patients younger than 16 years old were excluded.

#### Primary cohort

The primary cohort consists of patients in the first and second wave of infection from 1st January to 28th April 2020. To analyse the distribution of laboratory markers for different aetiology of pneumonia, patients that had nasopharyngeal aspirate (NPA) virologic sampling tested for common respiratory pathogens using multiplex PCR with or without sputum culture were selected. Patients were categorised into the following six disease groups: COVID-19, other viral pneumonia, bacterial pneumonia, clinical pneumonia, other infection, and other diseases. For patients included in COVID-19, other viral and bacterial pneumonia groups, they must be laboratory-confirmed positive by their respective laboratory tests. Viral and bacterial pneumonia is confirmed by either PCR or sputum culture. Patients that have partial laboratory tests or negative laboratory test results but has an ICD-9 classification of pneumonia were a group as clinical pneumonia. For other infection and disease, to ensure the patient does not have pneumonia pathogens, patient included to the groups must have negative test results for RT-PCR for SARS-CoV-2 and other common viral pathogens and sputum culture for bacterial infection. A detailed summary for cohort selects and lists of pathogens tested by PCR are listed in Supplementary Fig. 1.

#### Validation cohorts

To evaluate the performance of the modelling in discriminating the disease groups, the model was tested on three different validation cohorts across different time periods during the epidemics in Hong Kong. The first validation cohort (validation set 1) consisted of all COVID-19 patients presented in Hong Kong between 16th February to 2nd March with patients from 21 different hospitals. Negative patients for the validation set 1 were randomly sampled in the same period to give approximately 6% prevalence. To assess the generalisability of the findings, the second and third validation cohorts were obtained between 20th to 31st July 2020, which coincided with the third wave of local outbreak in Hong Kong. The second validation cohort (validation set 2) consisted of consecutive suspected patients presented across Hong Kong in 27 hospitals over 4 days between 20th to 23rd July, and the third validation cohort (validation set 3) was based on consecutive patients at a single hospital (XX Hospital) between 24th to 31st July. For validation set 1 and 3, in addition to laboratory test results, clinical details and frontal chest radiographs were also retrieved for analysis. Clinical details included travel or contact history, patient condition and symptoms at presentation, and were obtained from reviewing patient admission notes or discharge summaries.

### Statistical analysis

The patient demographics and the blood test results for haemoglobin, haematocrit, white blood cells (WBC), neutrophils, lymphocytes, monocytes, platelets, CRP and LDH were recorded and analysed for each disease group. For each variable, normality was tested by Shapiro-Wilks test. Comparison across diseases groups was tested by Kruskal–Wallis H test, with post hoc Mann–Whitney U test for statistical difference between individual groups. The effect size of laboratory markers between each group was estimated by the common language effect size f*.* f is equivalent to the area under the curve (AUC) for the receiver operating characteristic curve (ROC). Correlation between each test marker and age were also analysed by Spearman’s rank correlation coefficient r_s_.

### Modelling and evaluation

To analyse the discriminability of the laboratory markers, the features were modelled by machine learning to classify whether the patient has COVID-19, other viral pneumonia, bacterial pneumonia or non-pneumonia. The training set for the model was based on the patients from the primary cohort with overlapping patients from the validation sets removed. Patients that were classified as clinical pneumonia were not included in the modelling. The model was evaluated in the three validation sets to assess the performance and generalisability. In addition to the machine learning model (ML), the performance was compared with a clinical model and radiologist reads of frontal chest radiographs to provide a baseline for evaluation.

#### Machine learning model

To develop the ML model for classification of the diseases, several binary classification algorithms and classifiers were considered: Categorical gradient boosting (CatBoost), support vector machine (SVM), and logistic regression. Catboost is an open-source ensemble method based on gradient boosted decision tree designed for heterogeneous features types^35,36^. For SVM, gaussian, second-degree polynomial, and third-degree polynomial degree kernel function were tested. Each classifier was trained with 80% of the training set with the other 20% used for cross-validation, model selection, and threshold selection. To alleviate the problem of class imbalanced, a class-weighted cross-entropy loss was used as the loss function for all the tested classifers. For handling of missing values, the median feature value from the training set was used for the training of SVM and logistic regression. While no specific imputation is needed for the training of CatBoost as the optimal effect of missing values in the input are learned by CatBoost algorithm.

### Clinical model

A clinical model based on the blood test was devised. The model is based on the early observation that lymphopenia associated with COVID-19. Local diagnostic ranges for lymphocytes were used for the model. A patient is classified as likely to have COVID-19 if the patient has a lymphocytes count of less than 3.89 × 109/L and at least one of the following condition: (a) had close contact with a confirmed case, (b) had a travel history to an affected area classified as having active infections (e.g. mainland China, Europe and the US), (c) presented with fever (temperature > 37.5 °C), (4) required supplemental oxygen on admission.

#### Radiologist interpretation and combined radiologist ML model

A pre-defined set of CXR findings were used based on local experience and emerging literature to define “typical” radiographic features of COVID-19^13,17^. Radiologist interpretation of the frontal chest radiographs was performed on the validation set 1 and validation set 3. For validation set 1, four board-certified radiologists (2, 5, 10, and 15 years of experience) with subspecialty training in thoracic radiology read the films independently and blinded of RT-PCR results. The consensus agreement was used as the reference standard if two or more radiologists agreed on the finding. If there was a two-way tie, i.e. two radiologists reported positive finding, and two radiologists reported negative results, then the final prediction will be positive. This is because the aim is to increase sensitivity. For validation set 3, only one radiologist with thoracic radiologist read the films.

As most confirmed patients were admitted to hospital and owing to extensive testing and contact tracing, it is thought that a lot of patients were at the early stages of the disease. Chest radiographs may be normal, or if changes were present, they might be too subtle to be detectable. Hence, radiologist interpretation of chest radiographs alone will be unlikely to achieve very high sensitivity in detecting COVID-19. In order to maximise sensitivity for a combined ML model, the prediction of the model is deemed positive if either the ML model or radiologist reads positive (please refer to Supplementary Document for more details).

#### Evaluation

The AUC, accuracy, sensitivity, specificity, positive prediction value (PPV), and negative prediction value (NPV) were calculated for the prediction of each model. 95% confidence intervals (CI) for accuracy, sensitivity, and specificity were calculated using Clopper-Pearson “exact” methods^37^. Standard logit methods and Delong methods were used to estimate the CI for the predictive values and AUC, respectively^38,39^. In addition to the performances of the model, feature importance and interaction were analysed by using post-model Shapley additive explanations (SHAP) analysis^40^.

## Supplementary Information

Supplementary Information.

## Data Availability

Due to the retrospective nature of the study, specific patient level data used for this study cannot be made publicly available as patients did not agree for their data to be shared publicly. De-identified data may be available upon reasonable request.
