# Supplementary material for: Machine learning application for the prediction of SARS-CoV-2 infection using blood tests and chest radiograph
Source: Sci Rep. 2021 Jul 9;11:14250. doi: 10.1038/s41598-021-93719-2 (PMC8270945; doi:10.1038/s41598-021-93719-2)
Supplement: Supplementary file 1 — Supplementary Information. [file 41598_2021_93719_MOESM1_ESM.docx]

**Supplementary Document**

**Title:**

**Machine learning application for the prediction of SARS-CoV-2 infection using blood tests and chest radiograph**

**Author List**

Richard **Du**^1,2^, Efstratios D **Tsougenis**^2^, Joshua WK **Ho**^3^, Joyce KY **Chan**^4^, Keith WH **Chiu**^1^, Benjamin XH **Fang**^5^, Ming, Y **Ng**^1,6^, Siu-Ting **Leung**^7^, Christine SY **Lo**^5^, Ho-Yuen F **Wong**^5^, Hiu-Yin S **Lam**^5^, Long-Fung J **Chiu**^8^, Tiffany **So**^9^, Jeffrey KT **Wong^10^**, Yiu Chung I **Wong^11^**, Kevin **Yu^11^**, Yiu-Cheong **Yeung^12^**, Thomas **Chik^12^**, Joanna WK **Pang ^13^**, Abraham Ka-chung **Wai**^14^, Michael D **Kuo**^1^, Tina, PW **Lam**^5^, Pek-Lan **Khong^1^**, Ngai-Tseung **Cheung^15^**, Varut **Vardhanabhuti^1,^***

* Corresponding author

Corresponding Author Email: [varv@hku.hk](mailto:varv@hku.hk)

**Affiliations:**

1. Department of Diagnostic Radiology, Li Ka Shing Faculty of Medicine, The University of Hong Kong, Hong Kong SAR, China

2. Artificial Intelligence Lab, Information Technology and Health Informatics Division, Hospital Authority, Hong Kong SAR, China

3. The School of Biomedical Sciences, Li Ka Shing Faculty of Medicine, The University of Hong Kong, Hong Kong SAR, China

4. Clinical Systems, Information Technology and Health Informatics Division, Hospital Authority, Hong Kong SAR, China

5. Department of Radiology, Queen Mary Hospital, Hong Kong SAR, China

6. Department of Medical Imaging, The University of Hong Kong-Shenzhen Hospital, Shenzhen, China

7. Department of Radiology, Pamela Youde Nethersole Eastern Hospital, Hong Kong SAR, China

8. Department of Radiology and Imaging, Queen Elizabeth Hospital, Hong Kong SAR, China

9. Department of Imaging & Interventional Radiology, Faculty of Medicine, The Chinese University of Hong Kong

10. Department of Imaging & Interventional Radiology, Prince of Wales Hospital, Hong Kong SAR, China

11. Department of Radiology, Tuen Muen Hospital, Hong Kong SAR, China

12. Department of Medicine, Princess Margaret Hospital, Hong Kong SAR, China

13. Health Informatics, Information Technology and Health Informatics Division, Hospital Authority, Hong Kong SAR, China

14. Emergency Medicine Unit, Li Ka Shing Faculty of Medicine, The University of Hong Kong

15 Information Technology and Health Informatics Division, Hospital Authority, Hong Kong SAR, China

**Testing criteria by for COVID-19 testing in Hong Kong SAR, China**

Since the start of the surveillance beginning 1^st^ Jan 2020, the following criteria were used. Patients who:

i. Presented with fever or acute respiratory illness or pneumonia; and

ii. Either one of the following conditions within 14 days before onset of symptom:

• With travel history to a place with active community transmission of COVID-19 (including all places outside Hong Kong currently); or

• Had close contact with a confirmed case of COVID-19.

As of 6^th^ of April, the criteria broadened to include any patients with any acute symptom irrespective of the severity, types of symptoms or travel history.

One negative testing is required if no prior positive results were found. However, if the patient was found to be positive and undergo treatment, to be confirmed as negative, at least 2 samples of negative had to be detected. For more details of testing criteria and provision please refer to following publication^1^.

**Cohort Selection for Primary Cohort**

For patients included in COVID-19, other viral and bacterial pneumonia groups, they must be laboratory-confirmed positive by their respective laboratory tests. Viral and bacterial pneumonia is confirmed by either PCR or sputum culture. Patients that have partial laboratory tests or negative laboratory test results but has an ICD-9 classification of pneumonia were a group as clinical pneumonia. For other infection and disease, to ensure the patient does not have pneumonia pathogens, patient included to the groups must have negative test results for RT-PCR for SARS-CoV-2 and other common viral pathogens and sputum culture for bacterial infection.

Due to patients reviewed were from a large number of different hospitals in Hong Kong, different viral testing were conducted across patients. The virology test by PCR taken can be summarised in the the following three groups:

**Group 1:** Influenza A, Influenza B and Respiratory Syncytial Virus.

**Group 2**: all of above, Influenza C, Adenovirus, Enterovirus/Rhinovirus, Human metapnuomovirus, Parainfluenza 1 to 4.

**Group 3**: all of above, Coronavirus 229E, HKU1, NL63, OC43, Bordetella parapertussis, Bordetella pertussis, Chlamydia pneumoniae Mycoplasma pneumoniae.

In additional to the criteria of frontal chest x ray and complete blood counts test at diagnosis, to be included in the primary cohort patients must have atleast the abovemention virology test or sputum culture for bacterial infection. Patient with positive findings from the testing were included. To reduce uncertainty, to be included as negative patient or (other diseases), the patient must have taken for SARS-CoV-2, sputum culture, and at least group 1 and group 2 tests. Summary of the patient flow in the primary cohort selection is given in **Supplementary Figure 1.**


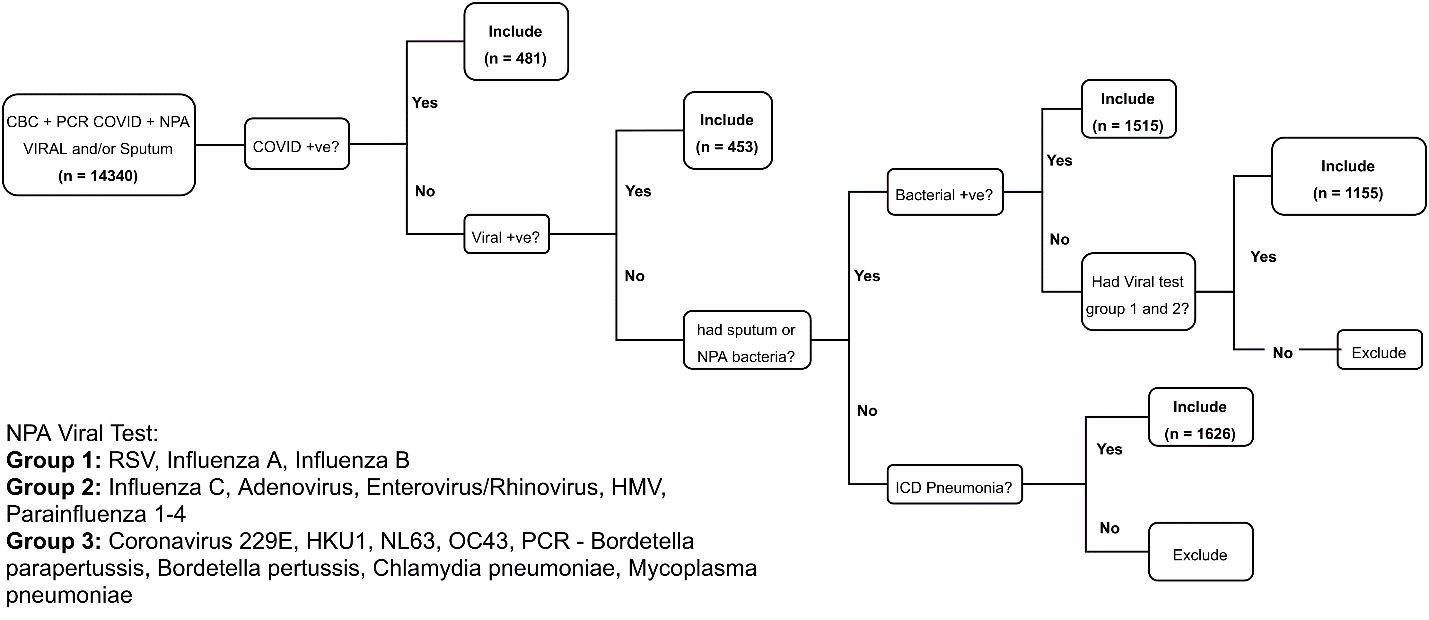


**Supplementary Figure 1.** Schematic and breakdown of patient flow for primary cohort selection.

**Combined and Chest Radiograph Model**

All confirmed patients were admitted to hospital and owing to extensive testing and contact tracing, it is thought that a lot of patients were at the early stages of the disease. Chest radiographs may be normal, or if changes were present, they may be too subtle to be detectable. Hence, radiologist interpretation of chest radiographs alone will be unlikely to achieve very high sensitivity in detecting COVID-19. The final prediction was computed based on the rule that if either the prediction for ML blood model or CXR was positive then final prediction was positive. The aim of this is to increase the sensitivity of the final model prediction. Thus rule-based algorithm was as followed:

ML blood (positive) + CXR (positive) then final prediction = **POSITIVE**

ML blood (positive) + CXR (negative) then final prediction = **POSITIVE**

ML blood (negative) + CXR (positive) then final prediction = **POSITIVE**

ML blood (negative) + CXR (negative) then final prediction = **NEGATIVE**

**Supplementary Tables**

**Supplementary Table 1.** Baseline demographics and laboratory characteristics of the validation set 1.

|  | **COVID-19** | **Other viral PNA** | **Bacterial PNA** | **Other** |
| --- | --- | --- | --- | --- |
| **Demographics** |  |  |  |  |
| Total, n | 40 | 49 | 45 | 62 |
| Female, n (%) | 19 (39) | 17 (38) | 29 (47) | 19 (39) |
| **Age, Years** |  |  |  |  |
| Mean ± SD | 63 ± 19 | 78 ± 13 | 63 ± 22 | 63 ± 19 |
| Median (IQR) | 65 (46 - 77) | 77 (72 - 87) | 65 (49 - 82) | 65 (46 - 77) |
| 16 - 35 | 0 (0) | 0 (0) | 0 (0) | 0 (0) |
| 35 - 50 | 5 (10) | 1 (2) | 9 (15) | 5 (10) |
| 50 - 65 | 9 (18) | 0 (0) | 8 (13) | 9 (18) |
| > 65 | 10 (20) | 6 (13) | 12 (19) | 10 (20) |
| **Haemoglobin g/dL** |  |  |  |  |
| Missing, n (%) | 0 (0) | 0 (0) | 0 (0) | 0 (0) |
| Mean ± SD | 11.8 ± 2.5 | 10.9 ± 2.9 | 11.4 ± 2.3 | 11.8 ± 2.5 |
| Median (IQR) | 11.8 (10.6 - 13.8) | 10.9 (9.1 - 12.8) | 12.0 (9.5 - 13.0) | 11.8 (10.6 - 13.8) |
| **Haematocrit** |  |  |  |  |
| Missing, n (%) | 0 (0) | 0 (0) | 0 (0) | 0 (0) |
| Mean ± SD | 0.4 ± 0.1 | 0.3 ± 0.1 | 0.3 ± 0.1 | 0.4 ± 0.1 |
| Median (IQR) | 0.4 (0.3 - 0.4) | 0.3 (0.3 - 0.4) | 0.4 (0.3 - 0.4) | 0.4 (0.3 - 0.4) |
| **WBC, 10^9^/L** |  |  |  |  |
| Missing, n (%) | 0 (0) | 0 (0) | 0 (0) | 0 (0) |
| Mean ± SD | 10.4 ± 8.1 | 14.1 ± 8.2 | 10.9 ± 5.5 | 10.4 ± 8.1 |
| Median (IQR) | 8.1 (5.5 - 10.9) | 13.1 (8.6 - 17.5) | 8.9 (6.7 - 13.6) | 8.1 (5.5 - 10.9) |
| **Lymphocyte, 10^9^/L** |  |  |  |  |
| Missing, n (%) | 4 (8) | 5 (11) | 0 (0) | 4 (8) |
| Mean ± SD | 1.1 ± 0.8 | 1.0 ± 0.8 | 1.3 ± 0.8 | 1.1 ± 0.8 |
| Median (IQR) | 0.9 (0.7 - 1.4) | 0.9 (0.6 - 1.2) | 1.1 (0.7 - 1.7) | 0.9 (0.7 - 1.4) |
| **Monocyte 10^9^/L** |  |  |  |  |
| Missing, n (%) | 4 (8) | 5 (11) | 0 (0) | 4 (8) |
| Mean ± SD | 0.7 ± 0.6 | 0.8 ± 0.4 | 0.7 ± 0.3 | 0.7 ± 0.6 |
| Median (IQR) | 0.6 (0.4 - 0.8) | 0.7 (0.4 - 1.0) | 0.6 (0.4 - 0.9) | 0.6 (0.4 - 0.8) |
| **Neutrophil 10^9^/L** |  |  |  |  |
| Missing, n (%) | 4 (8) | 5 (11) | 0 (0) | 4 (8) |
| Mean ± SD | 8.3 ± 7.6 | 12.1 ± 7.9 | 8.8 ± 5.8 | 8.3 ± 7.6 |
| Median (IQR) | 5.5 (4.3 - 8.8) | 10.5 (6.1 - 15.3) | 6.9 (4.6 - 11.6) | 5.5 (4.3 - 8.8) |
| **Platelet 10^9^/L** |  |  |  |  |
| Missing, n (%) | 0 (0) | 0 (0) | 0 (0) | 0 (0) |
| Mean ± SD | 227 ± 108 | 254 ± 112 | 253 ± 106 | 227 ± 108 |
| Median (IQR) | 221 (161 - 259) | 245 (182 - 334) | 241 (170 - 320) | 221 (161 - 259) |
| **CRP mg/dL** |  |  |  |  |
| Missing, n (%) | 19 (39) | 19 (42) | 32 (52) | 19 (39) |
| Mean ± SD | 6.6 ± 8.9 | 16.4 ± 11.0 | 7.4 ± 8.0 | 6.6 ± 8.9 |
| Median (IQR) | 3.5 (1.6 - 8.6) | 16.1 (9.0 - 22.0) | 5.9 (1.0 - 11.8) | 3.5 (1.6 - 8.6) |
| **LDH U/L** |  |  |  |  |
| Missing, n (%) | 33 (67) | 30 (67) | 41 (66) | 33 (67) |
| Mean ± SD | 263 ± 136 | 329 ± 180 | 234 ± 70.6 | 263 ± 136 |
| Median (IQR) | 230 (184 - 267) | 255 (208 - 435) | 212 (203 - 251) | 230 (184 - 270) |

**Supplementary Table 2.** Baseline demographics and laboratory characteristics of the training set.

|  | **COVID-19** | **Other viral PNA** | **Bacterial PNA** | **Other** |
| --- | --- | --- | --- | --- |
| **Demographics** |  |  |  |  |
| Total, n | 421 | 359 | 1431 | 847 |
| Female, n (%) | 186 (44) | 171 (48) | 537 (38) | 383 (45) |
| **Age, Years** |  |  |  |  |
| Mean ± SD | 41 ± 17 | 52 ± 22 | 74 ± 17 | 56 ± 22 |
| Median (IQR) | 39 (28 - 56) | 51 (33 - 69) | 78 (65 - 87) | 58 (36 - 74) |
| 16 - 35 | 0 (0) | 0 (0) | 0 (0) | 0 (0) |
| 35 - 50 | 170 (40) | 95 (26) | 45 (3) | 199 (23) |
| 50 - 65 | 110 (26) | 77 (21) | 83 (6) | 136 (16) |
| > 65 | 107 (25) | 74 (21) | 221 (15) | 174 (21) |
| **Haemoglobin g/dL** |  |  |  |  |
| Missing, n (%) | 0 (0) | 0 (0) | 0 (0) | 0 (0) |
| Mean ± SD | 14.0 ± 1.4 | 13.1 ± 2.2 | 11.1 ± 2.4 | 12.4 ± 2.5 |
| Median (IQR) | 14.0 (13.0 - 15.0) | 13.4 (12.0 - 14.6) | 11.1 (9.3 - 12.8) | 12.8 (11.1 - 14.3) |
| **Haematocrit** |  |  |  |  |
| Missing, n (%) | 0 (0) | 0 (0) | 0 (0) | 0 (0) |
| Mean ± SD | 0.4 ± 0.0 | 0.4 ± 0.1 | 0.3 ± 0.1 | 0.4 ± 0.1 |
| Median (IQR) | 0.4 (0.4 - 0.4) | 0.4 (0.4 - 0.4) | 0.3 (0.3 - 0.4) | 0.4 (0.3 - 0.4) |
| **WBC, 10^9^/L** |  |  |  |  |
| Missing, n (%) | 0 (0) | 0 (0) | 0 (0) | 0 (0) |
| Mean ± SD | 5.4 ± 1.8 | 8.8 ± 4.7 | 12.7 ± 13.1 | 10.9 ± 19.1 |
| Median (IQR) | 5.1 (4.2 - 6.2) | 7.8 (6.1 - 10.2) | 11.1 (7.6 - 15.3) | 8.6 (6.6 - 12.0) |
| **Lymphocyte, 10^9^/L** |  |  |  |  |
| Missing, n (%) | 4 (1) | 44 (12) | 87 (6) | 60 (7) |
| Mean ± SD | 1.3 ± 0.6 | 1.5 ± 0.9 | 1.1 ± 2.2 | 1.5 ± 2.3 |
| Median (IQR) | 1.3 (0.9 - 1.7) | 1.3 (0.8 - 1.9) | 0.9 (0.5 - 1.4) | 1.3 (0.8 - 1.9) |
| **Monocyte 10^9^/L** |  |  |  |  |
| Missing, n (%) | 4 (1) | 44 (12) | 90 (6) | 61 (7) |
| Mean ± SD | 0.5 ± 0.2 | 0.6 ± 0.4 | 0.7 ± 1.0 | 0.7 ± 0.5 |
| Median (IQR) | 0.5 (0.3 - 0.6) | 0.6 (0.4 - 0.8) | 0.6 (0.4 - 0.9) | 0.5 (0.4 - 0.8) |
| **Neutrophil 10^9^/L** |  |  |  |  |
| Missing, n (%) | 4 (1) | 44 (12) | 87 (6) | 60 (7) |
| Mean ± SD | 3.5 ± 1.6 | 6.7 ± 4.7 | 10.4 ± 7.5 | 8.0 ± 5.7 |
| Median (IQR) | 3.2 (2.4 - 4.2) | 5.4 (3.9 - 7.8) | 9.0 (5.9 - 13.5) | 6.2 (4.3 - 9.8) |
| **Platelet 10^9^/L** |  |  |  |  |
| Missing, n (%) | 0 (0) | 0 (0) | 4 (0) | 0 (0) |
| Mean ± SD | 219.1 ± 70.9 | 233.6 ± 86.1 | 247.5 ± 118.0 | 249.7 ± 101.7 |
| Median (IQR) | 204 (170 - 254) | 223 (176 - 279) | 232 (172 - 308) | 235 (186 - 296) |
| **CRP mg/dL** |  |  |  |  |
| Missing, n (%) | 47 (11) | 163 (45) | 573 (40) | 377 (45) |
| Mean ± SD | 1.6 ± 3.3 | 4.8 ± 7.5 | 9.7 ± 9.4 | 5.7 ± 8.4 |
| Median (IQR) | 0.4 (0.1 - 1.4) | 1.7 (0.3 - 5.8) | 7.1 (2.1 - 14.7) | 1.6 (0.2 - 8.3) |
| **LDH U/L** |  |  |  |  |
| Missing, n (%) | 30 (7) | 220 (61) | 886 (62) | 448 (53) |
| Mean ± SD | 206 ± 72.8 | 234 ± 130 | 365 ± 624 | 271 ± 446 |
| Median (IQR) | 184 (157 - 233) | 210 (165 - 256) | 252 (193 - 350) | 200 (168 - 266) |

**Supplementary Table 3.** AUC performance in classifying COVID-19 by different classifiers on the hold-out validation set. The median feature values of the training data were used for the missing values for both training and testing of the support vector machine classifier (SVM) and logistic regression.

| **Model** | **Positive/total** | **AUC** |
| --- | --- | --- |
|  | n | % |
| CatBoost | 80/612 | 94.6 |
| SVM – Gaussian Kernel | 80/612 | 76.6 |
| SVM – 3^rd^ Polynomial | 80/612 | 87.2 |
| SVM – 2^rd^ Polynomial | 80/612 | 73.9 |
| Logistic Regression | 80/612 | 88.2 |

**Supplementary Table 4.** Individual radiologist performance detecting COVID-19 in validation set 1 and validation set 3. An intraclass correlation (ICC) of 0.5 were obtained between radiologist reads.

|  | **Positive/total** | **Accuracy** | **Sensitivity** | **Specificity** | **PPV** | **NPV** |
| --- | --- | --- | --- | --- | --- | --- |
|  | **n** | **% (95% - CI)** | **% (95% - CI)** | **% (95% - CI)** | **% (95% - CI)** | **% (95% - CI)** |
| **Validation Set 1** |  |  |  |  |  |  |
| Radiologist 1 | 40/605 | 67.4 (63.5 - 71.2) | 65.0 (48.3 - 79.4) | 67.6 (63.6 - 71.5) | 12.4 (8.0 - 16.9) | 6.5 (94.6 - 98.3) |
| Radiologist 2 | 40/605 | 86.8 (83.8 - 89.4) | 25.0 (12.7 - 41.2) | 91.2 (88.5 - 93.4) | 16.7 (7.2 - 26.1) | 94.5 (92.6 - 96.4) |
| Radiologist 3 | 40/605 | 81.5 (78.2 - 84.5) | 25.0 (12.7 - 41.2) | 85.5 (82.3 - 88.3) | 10.9 (4.5 - 17.2) | 94.2 (92.1 - 96.2) |
| Radiologist 4 | 40/605 | 62.5 (58.5 - 66.4) | 62.5 (45.8 - 77.3) | 62.5 (58.3 - 66.5) | 10.5 (6.6 - 14.5) | 95.9 (93.9 - 97.9) |
| **Validation Set 3** |  |  |  |  |  |  |
| Radiologist 1 | 27/382 | 92.3 (89.1 - 94.8) | 53.8 (33.4 - 73.4) | 95.1 (92.3 - 97.1) | 45.2 (27.6 - 62.7) | 96.5 (94.6 - 98.5) |


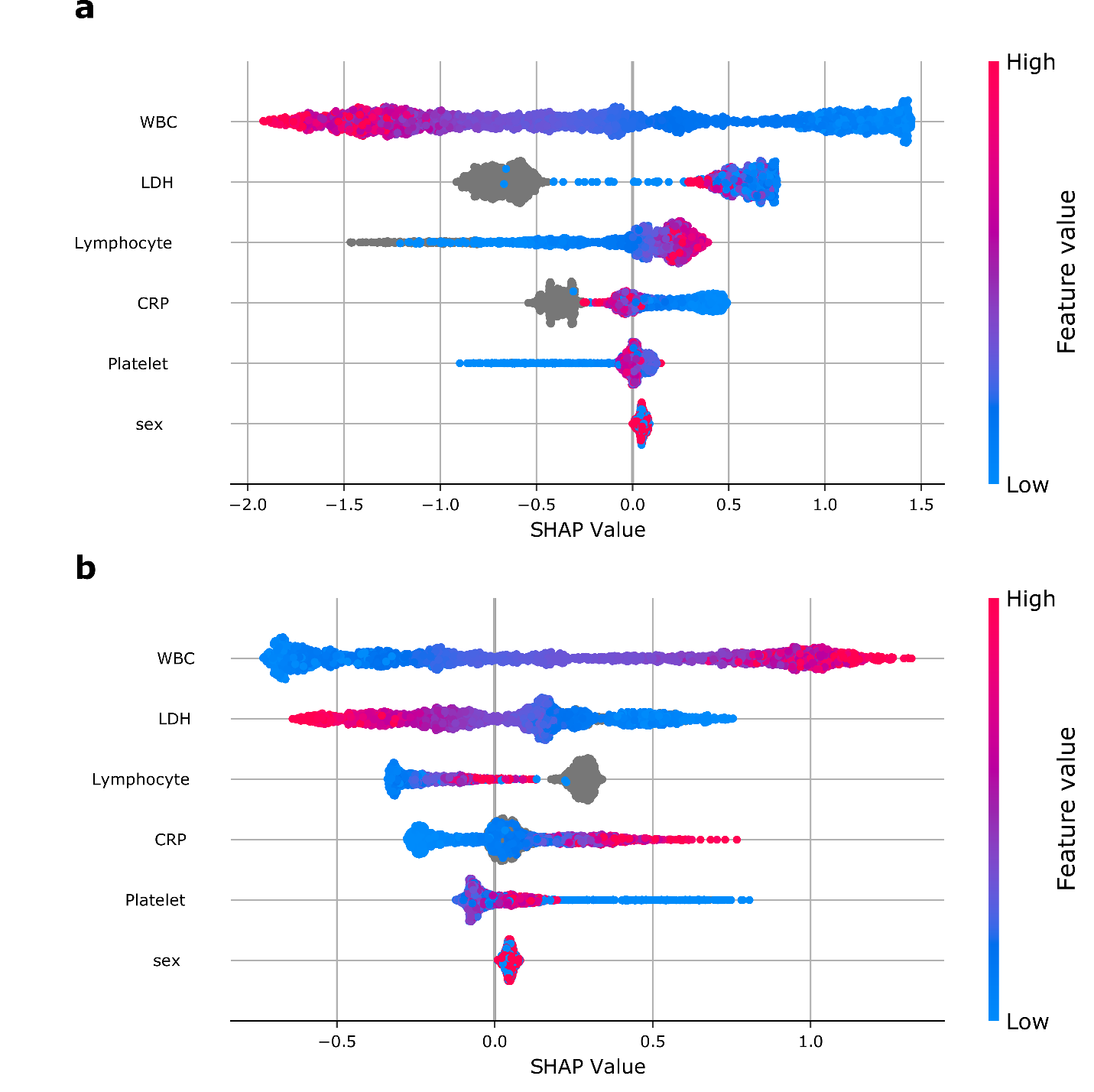


**Supplementary Figure 2**. SHAP summary plot of feature impact on prediction score of COVID-19 and bacterial pneumonia. The grey points represent impact given in case of missing values. (a) Summary plot of model impact on COVID-19 prediction. (b) Summary plot of model impact on bacterial pneumonia prediction.
